# Bilingualism, Dementia, and the Neurological Mechanisms in Between: The Need for a More Critical Look Into Dementia Subtypes

**DOI:** 10.3389/fnagi.2022.872508

**Published:** 2022-03-31

**Authors:** Yan-Yi Lee

**Affiliations:** Faculty of Education, University of Cambridge, Cambridge, United Kingdom

**Keywords:** bilingualism, the bilingual advantage debate, dementia, dementia subtypes, neurological mechanisms, pathology

## Introduction

With the continuation of the bilingual advantage debate, an outstanding question is whether bilingualism still carries any weight in dementia studies, and how it should be interpreted from here on. This opinion paper commences by justifying why the psycholinguistically-informed bilingual advantage debate, with its issues being more methodological in nature, should not impede the continuous neuroscientific efforts that explore the bilingualism-dementia link. In neuroscience, imaging techniques bring forth a different biological perspective, focusing more on the physical consequences of bilingualism in the brain rather than establishing a dichotomous argument over whether or not bilingualism is useful. Moving forward, what we currently know about the neurological mechanisms of bilingualism and dementia is still generic at present, and the lack of consideration of dementia's heterogeneity could slow down research progress on the bilingualism-dementia link.

To that end, this paper proposes a look into bilingualism and dementia subtypes, justifying the helpfulness of starting with specific areas of investigation: (i) why subtypes of dementia appear to be deferred by different lengths among bilinguals, (ii) how bilingual neural reserve interacts with the pathologies of dementia subtypes, and (iii) how bilingual neural reserve interacts with affected brain areas of dementia subtypes. These insights enable a more sophisticated understanding of bilingualism and its effects on the diverse mechanisms of memory loss and executive dysfunction, allowing us to understand how bilingualism compares with other types of mentally stimulating activities in old age.

## The Bilingual Advantage Debate and Dementia

The documentation of a delayed onset of dementia among bilinguals (Bialystok et al., 2007) has since ignited scholarly conversations on the bilingual advantage hypothesis. The psycholinguistic explanation to this is that the demands of bilingualism call for greater use of working memory (Grundy and Timmer, 2016), task switching (Prior and MacWhinney, 2010), and inhibitory control (Hartanto and Yang, 2019)—subsets of executive functions assumed to play a role in fostering the brain's cognitive reserve in old age (Guzmán-Vélez and Tranel, 2015). The bilingual advantage hypothesis is not unchallenged, however, as some empirical researchers failed to observe a statistically significant bilingual cognitive advantage in their studies, even for professional simultaneous interpreters (Paap, 2019; Ferreira et al., 2020). Reasons accounting for this contradiction are manifold:

Firstly, bilingualism itself is a complex phenomenon and hard to define strictly, being more of a continuous variable than a dichotomous one (Ferreira et al., 2020). As Heredia et al. (2020) caution, research has not yet firmly established the moderators and mediators in the bilingual benefits leading to cognitive reserve, which can be empirically problematic. The relationship between bilingualism and neuroprotection can be direct or indirect, and indirect factors are often confounding factors relevant to mental deficits in old age (such as education and intelligence). Qualitatively, bilingualism holds different statuses across contexts, which affects how the confounding factors turn out (e.g., in some geographical contexts, bilingualism is likely associated with higher levels of formal education, but in others, it could be associated with lower levels of formal education). Quantitatively, these confounding variables are challenging to control statistically with ANCOVA (unless studies include adequately large sample sizes), which can explain inconsistencies in findings (Paap, 2019). Further, conceptualizations of executive functions (Valian, 2016) and differences in how executive functions are operationalized in tasks could account for difficulties in replicating results. We should also consider how there may be more dimensions of cognitive benefits that we do not yet know to test among bilinguals. Teubner-Rhodes (2020) new concept of cognitive persistence in bilingualism– efforts made to optimize performance on mentally stimulating tasks– is one such example.

In psycholinguistics, the bilingual advantage debate may never truly be resolved for conceptual-methodological reasons, but that in itself should not discourage neuroscientists from further exploring the bilingualism-dementia link. Beyond psychometric limitations, few would deny that bilingualism is a mentally stimulating activity, and an investigation into its effects on the aging brain is promising nonetheless. As Vinerte et al. (2019) argue, behavioral studies may not be sensitive enough to test the advantages of bilingualism in the aging brain to a full extent, and neuroimaging data may complement our understanding of this area. As far as neuroimaging evidence is concerned, more than a hundred studies have already documented neuroanatomical changes in bilingual brains, inspiring preliminary discussions on their implications for pathology (see Taylor et al., 2022). In neuroscience, hence, we benefit from focusing more on the physical consequences of bilingualism in the brain and looking into what those changes entail for the development and progression of dementia.

## A Brief Summary and Critique of What Is Known So Far About the Neurological Mechanisms of Bilingualism and Dementia

Perani and Abutalebi (2015) were among the first to provide a review on the neurological mechanisms accounting for dementia delay in bilingual brains. The pair postulated that attentional and executive control are stimulated when engaging in bilingual activities, which in turn activates the mechanism of neural compensation and neural reserve. They also concluded in their review that bilinguals tend to develop functional connectivity, particularly in the fronto-parietal control network, and that bilingualism generally is associated with changes in white and gray matter density that may be protective against atrophy in old age. Kim et al. (2019) took the initiative to expand the investigation, screening a systematic set of relevant papers and pinpointing the functional and structural bilingual brain changes that converge across studies. Their findings largely echo those of Perani and Abutalebi's, confirming that bilingualism strengthens functional connectivity, increases gray matter density, and preserves white matter integrity. The authors also proposed that bilingual activity, with a strenuous need to attend to multiple aspects of language in simultaneous fashion (such as phonetics, grammar, and vocabulary), facilitates adult neurogenesis and synaptogenesis. From a neurobiological standpoint, it was also found by Perani et al. (2017) that bilingualism promotes metabolic connectivity.

The discussion on bilingualism and its neurological implications for dementia is still at its infancy, and the spotlight is cast on dementia more broadly. Of interest here is to examine whether the observed functional-structural changes involve regions of the brain that mediate bilingual processing, or the entire bilingual brain more broadly. Another point to consider is that dementia, much like aphasia, is heterogeneous in nature (Vega-Mendoza et al., 2019); investigating the bilingualism-dementia link only on a generic level may limit our outlook on bilingual stimulation and its relation to the diverse mechanisms of memory loss and executive dysfunction. It will hence be worthwhile to engage bilingualism research more meaningfully with dementia subtypes, through three specific avenues:

## Area to Explore (1): It Remains Unclear Why Bilingualism Appears to Defer Different Subtypes of Dementia by Different Lengths of Time

The current neurological knowledge that we have regarding the bilingualism-dementia link is fundamental, but looking beyond this, another critical question remains overlooked: in papers that observe a deferred onset of dementia in bilinguals, why are different subtypes of dementia delayed by different lengths of time?

Although we generally observe an encouraging correlation between being bilingual and having a delayed onset of dementia of 4–5 years, we should bear in mind that bilingualism does not defer each subtype of dementia to the same extent. The statistics may intrigue our curiosity further. Alladi et al. (2013) found a 3.2-year delay, a 6.0-year delay, and a 3.7-year delay among bilinguals for Alzheimer's dementia (AD), frontotemporal dementia (FTD), and vascular dementia (VaD), respectively. The delay was shorter for dementia with Lewy bodies (DLB), at 2.3 years, although it was mentioned that statistical significance was not reached in this case. The statistics reveal an even more alarming picture for semantic dementia, for which the onset was delayed for merely 0.5 years on average for bilinguals (Alladi et al., 2017). This finding is particularly striking, given that semantic dementia is a subtype of FTD, yet FTD in a general sense sees an average delay of at least 4–5 years in bilinguals. These differences are not yet accounted for in depth.

## Area to Explore (2): Structural and Functional Changes In Bilingual Brains and Their Interactions With the Etiologies and Pathologies of Dementia Subtypes

The studies we have concerning the dementia-bilingualism link so far are largely correlational (e.g., statistical studies examining whether bilingualism leads to a deferred onset of dementia) and inductive (e.g., examining functional-structural changes in the bilingual brain and inferring how bilingual activities might have led to those changes). What awaits investigation is how the structural and functional changes observed in bilingual brains interact with the etiologies and pathologies of the known dementia subtypes, as the causes and progressive pathways differ for each of them. For instance, how should we interpret the helpfulness of a strengthened bilingual brain in combating the effects of abnormal protein folding in Alzheimer's and abnormal protein deposits in dementia with Lewy bodies? How would being bilingual contribute to deferring the onset of vascular dementia than, say, maintaining a well-balanced lifestyle? A deeper look into this could reveal whether bilingualism does actually delay the onset of dementia entirely, or whether it merely creates a neural compensatory mechanism strong enough for dementia symptoms to manifest later.

## Area to Explore (3): The Areas of the Brain Affected In Subtypes of Dementia and Their Interactions With the Structural and Functional Changes In Bilingual Brains

As we understand it, subtypes of dementia may affect distinct areas of the brain: in Alzheimer's, neurons are typically destroyed in the entorhinal cortex and hippocampus (Meda et al., 2013); vascular dementia patients suffer from destroyed white matter beneath the cortex (Hase et al., 2018); dementia with Lewy bodies affects a variety of areas including the cerebral cortex (Obi et al., 2008), the hippocampus, the medial temporal lobe and so forth (Oppedal et al., 2019). Frontotemporal dementia involves atrophy in the frontal and temporal lobes; and in semantic dementia particularly, the anterior inferior temporal lobe is heavily affected (Chan et al., 2001). Coupled with this, it is also necessary to acknowledge that bilingual activity, depending on what skill and complexity it entails (e.g., reading in a linguistically distant language, speaking in a linguistically similar language, engaging in intense simultaneous interpretation), involves different areas within the brain. The emerging inquiry here is whether the diverse kinds of bilingual activity, which use up different cognitive demands, may strengthen the neural reserve of different areas in the brain. By way of illustration, is it possible that an intense intervention of simultaneous interpreting (Dong and Zhong, 2019) will fortify task switching and inhibitory control—activities that take place in the frontotemporal lobe– to such a point that the onset of frontotemporal dementia may get delayed even further? Or, would it be right to conjecture that continuous foreign vocabulary learning, which shapes gray matter structure in the hippocampus (Bellander et al., 2016), may lessen the effects of hippocampal decline in Alzheimer's disease? Here, an assumption to examine with caution is that increased activity in bilingualism-related brain regions would naturally strengthen functional connectivity and neurogenesis in those given regions.

## Discussion

The intersection of bilingualism and dementia is challenging to navigate due to the highly dynamic nature of both phenomena. Scholars concur that there is not one single cognitive outcome of bilingualism (Kroll and Chiarello, 2015), and neither should dementia be seen as one unitary disease given its heterogeneity. Further, bilingualism is not the only factor said to contribute to the delay of dementia, and this may complicate investigations into the bilingualism-dementia link.

Despite these complexities, there is reason to believe that this line of inquiry is worth investigating. Firstly, the tripartite link between (i) bilingual cognitive demand, (ii) anatomical-functional changes of the brain, and (iii) pathological bases of dementia subtypes could perhaps open the gateway for us to understand why bilingualism defers dementia by a period of 4 to 5 years in a general sense (Why not more? Why not less?). It could shed light on the affordances and limitations of bilingualism in its role in delaying dementia subtypes (e.g., would more intense bilingual code switching strengthen the frontotemporal lobe and what are its consequences for dementia delay? Bilingualism is not omnipotent (Bak and Alladi, 2014), but what exactly are its limitations in delaying cognitive decline?). In a similar vein, it may also open the gateway to understand how bilingualism, as a dementia-delaying activity, compares with other mentally stimulating activities that have also been scientifically proven to delay the onset of dementia in general, such as playing an instrument (Hanna-Pladdy and MacKay, 2011) or engaging in exercise (Colcombe and Kramer, 2003). Taken together, these questions could lead the way to uncover cognitive stimulation and dementia on a broader basis and assist with interventional planning more effectively.

## Author Contributions

The author confirms being the sole contributor of this work and has approved it for publication.

## Conflict of Interest

The author declares that the research was conducted in the absence of any commercial or financial relationships that could be construed as a potential conflict of interest.

## Publisher's Note

All claims expressed in this article are solely those of the authors and do not necessarily represent those of their affiliated organizations, or those of the publisher, the editors and the reviewers. Any product that may be evaluated in this article, or claim that may be made by its manufacturer, is not guaranteed or endorsed by the publisher.
